# Ethnic Minorities’ Impression Management in the Interview: Helping or Hindering?

**DOI:** 10.3389/fpsyg.2017.00086

**Published:** 2017-02-01

**Authors:** Eva Derous

**Affiliations:** Department of Personnel Management, Work, and Organizational Psychology, Faculty of Psychology and Educational Sciences, Ghent UniversityGhent, Belgium

**Keywords:** impression management, interview, ethnic minorities, professional experience, ethnic identification, social dominance orientation

## Abstract

Cross-cultural impression management (IM) has not been considered much, which is remarkable given the fast rate at which the labor market is becoming multicultural. This study investigated whether ethnic minorities and majorities differed in their preference for IM-tactics and how this affected ethnic minorities’ interview outcomes. A preliminary study (focus groups/survey) showed that ethnic minorities (i.e., Arab/Moroccans) preferred ‘entitlements’ whereas majorities (i.e., Flemish/Belgians) preferred ‘opinion conformity’ as IM-tactics. An experimental follow-up study among 163 ethnic majority raters showed no main effect of IM-tactics on interview ratings. Ethnic minorities’ use of IM-tactics only affected interview ratings if rater characteristics were considered. Specifically, interview ratings were higher when ethnic minorities used opinion conformity (i.e., majority-preferred IM-tactic) and lower when minorities used entitlements (i.e., minority-preferred IM-tactic) if recruiters were high in social dominance orientation, and when they felt more experienced/proficient with interviewing. IM-tactics are a human capital factor that might help applicants to increase their job chances on the labor market. It is concluded that ethnic minority applicants’ preferences for certain IM-tactics might lead to bias even in structured interview settings, but that this depends on ethnic majority recruiters’ interview experience and ingroup/outgroup attitudes. Implications for research and practice are discussed.

## Introduction

Employment interviews have always been and still are one of the most frequently used selection tools around the world (Macan, 2009), and often, even the only tool organizations use to screen applicants (Kristof-Brown et al., 2002; Levashina et al., 2014). Given the importance of the interview in the application process, applicants try their very best to impress interviewers through the use of impression management (IM) tactics in the employment interview. Applicants’ IM^1^ has been defined as a goal-directed conscious or unconscious process, in which applicants attempt to influence perceptions of recruiters/interviewers by regulating and controlling the information they exchange in the interview (Barrick et al., 2009). Not surprisingly, many studies have been conducted for a better understanding of applicants’ IM-tactics on work-related outcomes and interview outcomes in specific (Higgins et al., 2002; Barrick et al., 2009). Studies, for instance, have investigated dispositional antecedents of IM (like applicants’ personality; Kristof-Brown et al., 2002; Van Iddekinge et al., 2008; Bourdage et al., 2015), types of IM-behaviors used by applicants (like verbal vs. non-verbal behaviors; self-focused vs. other-focused tactics; deceptive vs. non-deceptive tactics; McFarland et al., 2003; Levashina and Campion, 2006; Roulin et al., 2015), effects of the use of IM-tactics on interview outcomes (Barrick et al., 2009; Proost et al., 2010), personal and situational factors that moderate the effect of IM-tactics on interview outcomes (like applicants’ self-esteem, interview structure and length; e.g., Ellis et al., 2002; Tsai et al., 2005; Peeters and Lievens, 2006; Chen et al., 2011; Levashina et al., 2014), and interviewers’ sensitivity to IM-tactics in structured interviews (e.g., Lievens and Peeters, 2008).

However, despite the abundance of research on IM effects, its determinants and moderators, very few studies have paid attention to *cultural differences in IM use* and *effects* on interview outcomes (Tsai and Huang, 2013; Bolino et al., 2016). This is remarkable given the fast rate at which the labor market is becoming multicultural and organizations search for talented workers, also among previously unexplored talent groups, like ethnic minorities. However, in Western-Europe, ethnic minority applicants still suffer lower labor market outcomes when compared to equally qualified applicants from ethnic majority groups (OECD, 2008, 2015). Both human capital factors (e.g., language proficiency, educational level, etc.; De Meijer et al., 2007; Hiemstra et al., 2013) and biased decision-making (e.g., the use of cognitive scripts, stigmatization, and prejudiced reactions; Derous et al., 2016) may explain the observed differences to some extent.

The first goal of this study was to investigate a human capital factor that has not been considered much when evaluating ethnic minorities’ job chances, namely ethnic minorities’ use of IM-tactics during the employment interview. IM-tactics are typically used by applicants to enhance their job chances but whether they do so might depend on the type of IM-tactics applicants use. Specifically, we investigated whether ethnic minorities and majorities differ in their preference for IM-tactics and whether that affect any discrepancy in interview outcomes. Second, and with a few exceptions (Delery and Kacmar, 1998; Lievens and Peeters, 2008), little studies have investigated *recruiter characteristics* as potential moderators of IM-tactics on interview outcomes, let alone interactions with applicants’ ethnic background and preferred IM-tactics. More research, however, is needed to understand *why* IM impacts recruiter ratings (Levashina et al., 2014; Bolino et al., 2016). Hence, as a second goal, this study considered ethnic majority recruiter characteristics (i.e., ingroup/outgroup attitudes; professional experience) as potential moderators of ethnic minority applicants’ use of IM-tactics on interview outcomes.

In the next paragraph, we first discuss why one can expect cultural differences in the use of IM-tactics (i.e., based on the Cross Cultural Impression Management Discourse model; Bilbow, 1997). We further consider why such differences may result into lower interview outcomes, and what we already know about cultural differences in the use of IM-tactics in hiring contexts. Next, we investigate how cultural differences in the use of IM-tactics might interact with recruiter characteristics (i.e., prejudiced ingroup/outgroup attitudes; interview experience) in explaining ethnic minorities’ interview outcomes.

## Cultural Differences in Impression Management

### Cross-Cultural Impression Management

Bilbow (1997) formulated the Cross Cultural Impression Management Discourse model (CCIM model^1^), in which a person’s sociocultural background is expected to affect both the use and interpretation of IM-tactics in organizations. The CCIM model considers IM as a central aspect of dialog in which speakers (e.g., applicants) project impressions of themselves to others (e.g., recruiters), and receivers attribute characteristics to speakers on the basis of their discourse. Although the use of IM-tactics seems quite universal (Barrick et al., 2009), the central tenet of Bilbow’s CCIM model is that its concrete manifestation may be different for different individuals in diverse situations. Bilbow’s CCIM discourse model particularly states that both speakers’ use of IM-tactics and receivers’ attribution processes are affected by the features of one’s sociocultural environment, including factors such as speakers and receivers’ ethnic background, social status, and sociocultural norms and values.

It is further assumed that when people have a common sociocultural background (like the same ethnic background), “there may be a significant degree of similarity between the impressions speakers believe their discourse to be projecting and hearers’ perceptions of speakers,” which will lead to more ‘resonance’ (Bilbow, 1997, p. 465). Hence, when recruiters and applicants share a common background, one can expect them to also share similar ideas regarding the appropriateness and effectiveness of the use of IM-tactics in a particular social situation, like the employment interview.

However, Bilbow (1997) also assumes that the degree of resonance will be substantially less in cross-cultural situations: when recruiters and applicants do not share a common background, they may not share similar ideas regarding the appropriateness and effectiveness of IM-tactics. As a consequence, there is a possibility that raters from one culture may not appreciate IM-tactics used by others (Paulhus et al., 2013). In line with this, Horverak et al. (2013) showed that Turkish immigrant applicants who expressed cultural maintenance preferences in their private life (e.g., spare time) received lower hirability ratings by Norwegian managers who saw videotaped interviews of these candidates when compared to equally qualified Turkish immigrant applicants who appeared to be more assimilated. Moreover, deviations from any cultural norm may stress ingroup/outgroup differences. Social categorization and identity theory (Tajfel and Turner, 1986) further posits that strongly identified outgroup members may be more subject to outgroup derogation. That is, people’s need for a positive identity (self/group) and their tendency to protect a positive social identity may instigate ingroup favoritism to the detriment of outgroup members. Hence, ethnic minority applicant who use IM-tactics that deviate from the ones that are typically used or preferred by ethnic majority recruiters (i.e., ‘minority-preferred IM-tactics’), may receive lower appreciation (like interview ratings) than those who use IM-tactics that do not deviate from the ones that are typically preferred (and expected) by ethnic majority raters (i.e., ‘majority-preferred IM-tactics’).

What about not using IM-tactics, would that be more beneficial to ethnic minorities than using minority-preferred IM-tactics? One cannot not communicate (Watzlawick et al., 1967): also the absence of any action (like not using IM-tactics) has the potential to be interpreted as having some meaning. Not using IM-tactics could signal lower levels of assimilation and hence be as effective as using minority-preferred IM-tactics. Alternatively, one can expect the use of minority-preferred IM-tactics to stress ethnic minority applicants’ outgroup status to an even larger extent than when ethnic minorities do not use any IM-tactics. Therefore, and although the use of IM-tactics are somewhat expected and even rewarded by recruiters (Higgins and Judge, 2004), using no IM-tactics might result in either the same or higher ratings than when ethnic minorities use IM-tactics that deviate from the cultural norm and accentuate their outgroup status.

### Cultural Differences in Applicants’ Use of IM-Tactics

A limited number of studies have investigated cultural differences in applicants’ use of self-presentation tactics in hiring contexts. Sandal and Endresen (2002), for instance, were among the first to show that Norwegian students had lower ‘good impression’ scores on the CPI personality inventory than American students in the ‘fake good condition,’ whereas differences were smaller in the ‘honest condition.’ König et al. (2011) further showed Icelandic and Swiss part-time business students to report less self-presentation tactics in selection procedures than Northern-American business students. Somewhat unexpectedly, König et al. (2012) found Chinese students to report similar frequencies of self-presentation than Northern-American students. Due to their high unemployment rates, Chinese students might have used more self-presentation than expected on the basis of the proposed Chinese modesty hypothesis. These findings support Bilbow’s idea that sociocultural background – including one’s status and economic position- may shape individuals’ engagement in IM (Bilbow, 1997; Zaidman and Drory, 2001).

As far as the employment interview concerns, international surveys conducted by Bye et al. (2011) and Sandal et al. (2014) also showed cross-cultural differences in university students’ envisioned self-presentation tactics. Self-presentation tactics were considered more important in countries with a strong cultural orientation (i.e., toward embeddedness, mastery, hierarchy) and with larger income disparities. Fell et al. (2016) further showed four of GLOBE’s nine cultural dimensions to relate significantly with employees’ attitudes toward dishonest IM in the employment interview (i.e., faking). Considering these findings, it is not unlikely that ethnic minority applicants impress recruiters in a different way than ethnic majority members.

To extend the sparse body of knowledge, this study focuses on IM-tactics preferred by Arab/Moroccan applicants^2^, a large and growing ethnic minority group in Western-Europe, whose members are at risk of downward assimilation (OECD, 2015). Arab/Moroccan job seekers, for instance, have the highest unemployment rates compared to other ethnic minorities and majorities in Belgium and Flanders in specific. Differences in sociocultural background may explain in part Arab/Moroccans’ lower labor market position (Bilbow, 1997). However, Southern-European and Mediterranean societies, like the Arab/Moroccan society, appear to be higher in collectivism than many Western-European societies, like Belgium/Flanders, being the context of this study (Hofstede, 2001; Phalet and Schönpflug, 2001). This may affect the way applicants impress recruiters: some suggest that members from individualistic-oriented societies may engage more in self-focused IM whereas members of collectivistic-oriented societies may engage more in other-focused IM (Phalet and Schönpflug, 2001; Dipboye et al., 2013). Hence, differential preferences and evaluations of IM-tactics among members from these societies can be expected^3^.

In sum, based on the CCIM discourse model and predictions from social categorization/identity theories, we investigated whether:

*Hypothesis 1:* Ethnic majority raters (i.e., Flemish/Belgian) will give lower ratings to ethnic minority applicants who use IM-tactics preferred by ethnic minorities (i.e., Arab/Moroccan) or no IM-tactics at all, than to equally qualified ethnic minority applicants who use IM-tactics preferred by the ethnic majority.

## Recruiter Characteristics and Impression Management

As the employment interview is a two-way process (Dipboye et al., 2013), recruiters’ characteristics and decision-making need to be considered when evaluating IM-tactics (Levashina et al., 2014). In his CCIM-model, Bilbow (1997) also suggests that raters’ attribution processes of perceived IM-tactics are affected by their sociocultural background. However, besides raters’ ethnic/cultural background, this framework is not very specific about rater characteristics that might affect attribution processes. We aimed to further explore this here. First, raters’ attribution processes and (biased) decision-making might be triggered by both stigmatizing information about a person belonging to an outgroup and one’s prejudiced attitudes toward members of that outgroup (e.g., Pryor et al., 2004). Hence, in line with social categorization/identity theory one can expect recruiters’ ingroup/outgroup attitudes to affect the interview evaluation if ethnic minority applicants use IM-tactics that deviate from the dominant, cultural norm. Further, biased decision-making is commonly believed to be driven by one’s cognitive scripts that are formed through and affected by one’s prior experiences and knowledge (Dipboye and Jackson, 1999; Derous et al., 2016). In the interview literature, however, it is less clear whether/how interviewer experience might impact the evaluation of stigmatizing information (e.g., ethnic information like minority-preferred IM-tactics). Therefore, this study also explored the role of interviewer experience on the effect of ethnic minorities’ use of IM-tactics on interview ratings. In the next paragraph, we first consider the potential moderating role of interviewers’ ingroup/outgroup attitudes in affecting biased interview outcomes, namely interviewers’ social dominance orientation (SDO) and ethnic identification. Their relevance and potential influence on the evaluation of IM-tactics are further discussed below. Thereafter, we turn our attention to recruiters’ experience in interviewing and evaluating applicants.

### Ingroup/Outgroup Attitudes

A first useful construct to understand the impact of ethnic information on individuals, is raters’ SDO. SDO^1^ is a psychological variable that refers to the extent one desires that one’s ingroup dominates and is superior to members of outgroups (Pratto et al., 1994). Umphress et al. (2007), for instance, showed that prospective employees of high-status groups (i.e., ethnic majorities) who were high in SDO, were more attracted to organizations composed of high-status, dominant employees. Furthermore, people high in SDO support group-differentiating ideologies, such as nationalism, cultural elitism, and racism, more so than people low in SDO (Pratto et al., 2000). In support of this, there is considerable evidence that SDO contains unique predictive value for prejudice and discrimination in many different settings and across many cultures (e.g., Ekehammar et al., 2004; Van Hiel et al., 2004). Ekehammar et al. (2004), for instance, showed SDO to correlate highly with racism, sexism, and prejudice against mentally disabled persons. SDO seems particularly associated with stereotype-based cognitive processing of information an –hence– biased judgments. For instance, in an experimental study, Goodwin et al. (1998) showed that high dominance interviewers neglected competency information about applicants and were more willing to hire the most sociable (instead of competent) applicant, whereas low dominance interviewers recognized the competent applicants and were more willing to hire these applicants than their less competent, sociable counterparts. Similarly, recruiters may be biased to favor members of their own social ingroup, depending on their degree of SDO (Pratto et al., 1994). Derous (2011), for example, showed in a scenario-based study that recruiters high in social dominance rated resumes of Arab/Moroccan applicants significantly lower than resumes of equally qualified ethnic majority applicants. Somewhat counterintuitively, however, authors have also argued that ethnic majority members high in SDO may be more comfortable with ethnic minorities who *maintain* their own cultural values and habits (like using minority-preferred IM-tactics) than those who *assimilate* (like using majority-preferred IM-tactics) and weaken group-based distinctions on which the system of dominance is built (i.e., ‘status boundary enforcement hypothesis,’ see Thomson et al., 2008; Guimond et al., 2010). Although some support has been found for the ‘status boundary enforcement hypothesis,’ empirical findings supporting this hypothesis are somewhat limited compared to the vast amount of empirical evidence showing ethnic majority members to have more positive attitudes toward immigrants who voluntarily adopt their language, cultural values and habits compared to those who do not (Van Oudenhoven et al., 1998). Because of this and in line with the limited research that has investigated the effects of ethnic majority recruiters’ SDO on ethnic minorities’ interview ratings (e.g., Cohrs and Asbrock, 2009; Derous, 2011), we assumed that ethnic majority recruiters high in SDO would express negative prejudice against Arab/Moroccan applicants who emphasize their cultural heritage by using minority-preferred IM-tactics.

*Hypothesis 2*: When compared to ethnic majority raters (i.e., Flemish/Belgian) who score low on SDO, those who score higher on SDO will give lower ratings to ethnic minority applicants who use IM-tactics preferred by ethnic minority members (i.e., Arab/Moroccan) or no IM-tactics at all, than to equally qualified ethnic minority applicants who use IM-tactics preferred by the ethnic majority.

A second and related individual difference variable that might moderate effects of culturally preferred IM-tactics on recruiters’ interview ratings, is recruiters’ ethnic identity (Phinney, 1996). Ethnic identity is part of one’s social identity and refers to feelings of ethnic belonging and pride, a secure sense of group membership, and positive attitudes toward members of one’s ethnic ingroup. Moreover, the greater one’s ethnic ingroup identification is, the more one may allocate social value to the ethnic ingroup rather than to the ethnic outgroup (Phinney and Ong, 2007). Ethnic identification has also been related to SDO in explaining outgroup derogation (Perry and Sibley, 2011; Moscatelli et al., 2016). However, people differ in the extent they identify themselves with their authentic ethnic ingroup and the degree to which this ethnic group is important and meaningful to them (Phinney and Ong, 2007). For instance, Arab/Moroccan minority applicants who use certain (minority-preferred) IM-tactics might stress their ethnic identity more than Arab/Moroccan minority applicants who do not. As a consequence, ethnic minorities who participate in activities of ethnic identity maintenance (e.g., by using minority-preferred IM-tactics) can be perceived as less assimilated and therefore, may be more likely to experience prejudiced treatment (Sellers and Shelton, 2003). Likewise, when ethnic majority recruiters identify themselves more strongly with their own ethnic ingroup, they may perceive Arab/Moroccan minority applicants as more dissimilar and more of a threat to their social identity, particularly when ethnic minorities use IM-tactics preferred by their own ethnic ingroup members. This prediction is in line with Bilbow’s CCIM-model that suggests raters’ attribution processes of perceived IM-tactics to be affected by raters’ sociocultural background. Whereas Bilbow (1997) did not investigate nor considered psychological mechanisms to explain this, the social identity theory (Tajfel and Turner, 1986) can be cited to substantiate that the more ethnic identity is aligned between ethnic majority recruiters and ethnic minority applicants, the better the evaluation of the ethnic minority applicant will be (Derous et al., 2009). Therefore, we expected that:

*Hypothesis 3*: When compared to ethnic majority raters (i.e., Flemish/Belgian) who less strongly identify with their ethnic ingroup, those who more strongly identify with their ethnic ingroup will give lower ratings to ethnic minority applicants who use IM-tactics preferred by ethnic minority members (i.e., Arab/Moroccan) or no IM-tactics at all, than to equally qualified ethnic minority applicants who use IM-tactics preferred by the ethnic majority.

### Interview Experience

According to Bilbow’s CCIM-model (1997), applicants’ discourses will also be ‘filtered’ by interviewers’ prior experiences and practices. It is, for instance, commonly assumed that experienced interviewers provide more valid ratings than less experienced interviewers, because experienced interviewers may have gained more professional insights throughout years of interviewing and may use less irrelevant information when evaluating applicants (Dipboye and Jackson, 1999). Remarkably, studies that have investigated professional experience considered ethnic majorities’ evaluations of ethnic majority applicants; how ethnic majority recruiters’ professional experience affect their judgments of ethnic minorities’ interview performances has not been investigated much. De Meijer et al. (2007), however, showed that experienced majority recruiters may use more irrelevant information when they judge ethnic minorities and, hence, risk more stereotypical decision-making when assessing ethnic minorities, which runs counter to what is commonly held to be true. Several other studies that have investigated interview experience –albeit not in the context of ethnic minority applicants– showed no beneficial effects (Lievens and Peeters, 2008) or even opposite effects of interview experience on information gathering and interview ratings (e.g., Gehrlein et al., 1993). For instance, Gehrlein et al. (1993) found that the criterion-related validity of college admission interviews was lower if interviewers were more experienced. Dipboye and Jackson (1999) discussed several studies that showed experienced interviewers not to be immune to the biasing effects of perceived similarity, attractiveness, and personal liking, which corroborates conclusions of De Meijer et al. (2007). Findings, however, seem far from conclusive (e.g., Marlowe et al., 1996; Posthuma et al., 2002), perhaps because of the way interviewers’ experience has been measured (Dipboye et al., 2013). Recruiters’ professional experience has typically been operationalized by the amount of interviews and/or time spent interviewing. However, quantifying interview experience in such a way may not reflect one’s perceived interviewing proficiency or expertise in conducting interviews, which can be expected to reflect professional experience in a more precise and comprehensive way than, e.g., ‘years of experience’ (Dipboye and Jackson, 1999).

Taken together, because majority interviewers may rely more on irrelevant information like cultural stereotypes (De Meijer et al., 2007) and because ethnic minorities who use minority-preferred IM-tactics may fit such cultural stereotypes more than those who use majority-preferred IM-tactics, one could expect from the CCIM-model even lower interview ratings for ethnic minority applicants who use minority-preferred IM-tactics. However, given the overall mixed predictions regarding effects of interview experience on valid interview judgments and the limited number of studies on its effect on ethnic minority applicants’ interview outcomes, we formulated the following research question to explore the potential role of ethnic majority raters’ (self-rated) interviewing experience in their evaluation of ethnic minorities’ use of IM-tactics.

*Research Question*: Will ethnic majority raters (i.e., Flemish/Belgian) who are more experienced still give lower ratings to ethnic minority applicants who use IM-tactics preferred by ethnic minority members (i.e., Arab/Moroccan) or no IM-tactics at all, than to equally qualified ethnic minority applicants who use IM-tactics preferred by the ethnic majority?

## Materials and Methods

### Ethics Statement

The study was carried out in accordance with the guidelines of the ‘General Ethical Protocol for Scientific Research at the Faculty of Psychology and Educational Sciences’ of the Ethical Commission of the Faculty of Psychology and Educational Sciences, which is the relevant university institutional review board that considers ethical aspects. In accordance with the Declaration of Helsinki, participants provided informed written consent prior to their participation. Participants were debriefed after all the data were collected.

### Participants

Participants were recruited through Belgian HR organizations, professional HR publications, HR students, and researchers’ own professional networks. In total, 550 potential participants were emailed the study link to participate of which 29.64% actually participated. As we aimed to measure ethnic majority reactions to ethnic minorities’ (i.e., Arab/Moroccan) use of IM-tactics during the employment interview, we had to remove two participants with an Arab/Moroccan descent. Hence, the final sample consisted of 163 Flemish/Belgian recruiters (also called ‘participants’ or ‘raters’) of which 71.2% (*n* = 116) women, and 50% (*n* = 83) having at least 2 years of professional experience in interviewing candidates at the selection stage.

### Design

An experimental study was conducted to test the hypotheses and the research question. The effects of IM-tactics (i.e., ‘entitlements’ vs. ‘opinion conformity’ vs. ‘no IM-tactics/control’) on job suitability ratings (i.e., the interview outcome) were investigated using a between-subjects design. Entitlements were preferred by members of the Arab/Moroccan minority group, whereas opinion conformity was preferred by the Flemish/Belgian majority group members (see Impression Management Tactics). We further investigated potential moderating effects of ethnic majority raters’ ingroup/outgroup attitudes (i.e., SDO; ethnic identification), and their professional experience with interviewing (i.e., self-rated interviewing proficiency) on the relation between the Arab/Moroccan applicant’s use of IM-tactics and job suitability ratings. The applicant’s ethnicity and gender were kept constant (i.e., male Arab/Moroccan applicant). Only male Arab/Moroccan applicants were considered because of their higher labor market participation compared to Arab/Moroccan females in Belgium (OECD, 2015), and because of the need to remove gender as a potentially confounding factor in the design (Sidanius and Veniegas, 2000).

### Procedure

Participants were sent an email explaining the study procedure as well as the voluntary nature of the study. In addition to providing one video-taped interview (see further), we also provided a context to make the focus on IM and (potential) hiring discrimination non-transparent. We specifically asked participants to participate in a study to optimize employment interviews. This information was repeated on the first page of the study website, on which participants also gave their informed consent.

Participants were first instructed to read the vacancy for a front-office position and the resume of a Moroccan male applicant. Thereafter, participants looked at a videotaped, structured employment interview that lasted 10 min. Participants were randomly assigned to one of the three IM-conditions in which they viewed the same interview script and the same Arab/Moroccan applicant, using another IM-tactic, as further explained below. Immediately after the interview, participants rated the applicant on job suitability (i.e., interview outcome). Next, participants completed measures of ethnic identification and SDO. They also provided personal background information (i.e., age, gender, nationality, ethnicity, professional experience). In the end, participants were thanked for their participation. Feedback (including debriefing) was provided when all data were collected.

### Impression Management Tactics

Since little is known about the kind of IM-tactics preferred by ethnic minority and majority applicants, we conducted an initial, exploratory study consisting of one focus group meeting and a follow-up survey to explore cultural differences in preference for IM-tactics (Krueger and Casey, 2000; Bachiochi and Weiner, 2002). As mentioned, we focused on one of the largest ethnic minority groups (i.e., applicants of Arab/Moroccan descent) in Western-Europe and Belgium/Flanders (with Flanders being the Northern part of Belgium) as the area of interest, in particular.

#### Focus Group

Following Krueger and Casey (2000), we selected participants who shared a common ethnic-cultural background to participate in the focus groups. This was done with the help of social workers of a ‘community development organization,’ that informs and helps ethnic minority members, e.g., with several employment-related issues. In total, nine Arab/Moroccan males, two facilitators and one neutral observer participated in the focus group meeting, which lasted 4 h. One of the facilitators (i.e., an employee of the community organization) shared the same ethnic-cultural background as the focus group participants; the other facilitator and observer were both members of the research team. Participants all agreed to participate in a voluntarily way. During the first part of the focus group meeting, participants role-played several employment interviews and IM-tactics were observed by the facilitators, the observer, and the other focus group participants. During the second part of the meeting, roleplays and IM-tactics were discussed in the focus group. Facilitators asked questions like “What did you tell the interviewer/recruiter to make a good impression on him/her?,” and “What is –according to you- the best way to impress the recruiter during the employment interview?”

The IM taxonomies of Stevens and Kristof (1995) and Ellis et al. (2002) were used as guidelines to evaluate the IM-tactics observed/mentioned. Two broad categories of IM-tactics have been distinguished in this literature, namely non-verbal IM-behavior (smiling, nodding, etc.) and verbal IM-behavior (Stevens and Kristof, 1995). Of interest to this study was only verbal IM-behavior, which is characterized by both defensive and assertive tactics (Ellis et al., 2002). Defensive tactics aim to protect or repair one’s image to avoid being negatively evaluated, like the use of excuses. Assertive tactics aim to acquire and promote favorable impressions to get positively evaluated. Such assertive tactics consist of both self-focused tactics like self-promotion and entitlements (i.e., to evoke attributions of competence by focusing the conversation on the applicant) and other-focused tactics like opinion conformity and other-enhancements (i.e., designed to evoke interpersonal attraction by focusing the conversation on the interviewer or rater).

Focus group results showed that Arab/Moroccan minority group members do use IM-tactics and –in particular– prefer assertive tactics like self-promotion and entitlements (no defensive tactics were mentioned). Examples are: “You have to say positive things about yourself. For example: I worked a lot and have already done a lot. I can do a lot of different things like...,” and “If you have some experience, you should definitely say this, and also that you have a lot of experience with many different tasks.” To further validate findings from the focus group as well as to investigate ethnic majorities’ preferences for IM-tactics, a follow-up survey was conducted (Morgan, 1997).

#### Survey

Participants were recruited via specific organizations (like Arab/Moroccan organizations) and researchers’ personal networks. A total of 53 respondents participated to the survey of which 32 were eligible (50% male; 50% Flemish). Participants indicated on a five-point Likert-type scale their preference for several IM-tactics, based on the IM taxonomies of Stevens and Kristof (1995) and Ellis et al. (2002). Survey results showed subtle differences in preferences for IM-tactics between ethnic minority (Arab/Moroccan) and majority (Flemish/Belgian) respondents. Arab/Moroccan participants (*M* = 3.63; *SD* = 0.88) preferred taking credits and claiming responsibility for positive outcomes (even if undeserved) more than Flemish respondents (e.g., “Do you prefer taking credits and claiming responsibility for positive outcomes during the job interview, even if not deserved by yourself?,” *M* = 3.06; *SD* = 0.77), *t*(30) = -1.92, *p* = 0.06, Cohen’s *d* = 0.71. Flemish participants (*M* = 3.56; *SD* = 0.81) preferred to express opinions and values they believed the recruiter would appreciate, more than Arab/Moroccan participants (e.g., “Do you prefer to express some opinions during the interview that you believe the recruiter/interviewer holds, to satisfy the recruiter/interviewer?,” *M* = 2.81; *SD* = 1.02), *t*(30) = 2.02, *p* = 0.05, Cohen’s *d* = 0.84.

Taken together, results of both the focus group and survey provided evidence that Arab/Moroccan participants prefer self-focused tactics like ‘entitlements,’ which means taking credit or claiming responsibility for positive outcomes even if that credit is undeserved. Ethnic majority participants (i.e., Flemish), on the other hand, rather preferred ‘opinion conformity’ or expressing values/beliefs/opinions that are known to be held (or can reasonably be assumed to be held) by the receiver/interviewer (Ellis et al., 2002). These results do not imply that Arab/Moroccans never use opinion conformity and Flemish applicants avert using entitlements. Results rather showed a pattern of preference in IM-tactics that depends on one’s ethnic background/community and that we aimed to further explore and validate in an experimental study. Therefore, these IM-tactics were included in different conditions of our experimental study, as is further explained below.

### Materials

#### Applicant

For the experimental study, we recruited an Arab/Moroccan confederate actor to act as the applicant, who spoke fluent Flemish (i.e., very similar to Dutch) without any Arab/Moroccan accent. The latter was requested in order not to induce any extra source of ethnic bias (i.e., as prompted by non-native accent; see Purkiss et al., 2006; Gluszek and Dovidio, 2010). Prior to recording the interviews, the confederate applicant received training to standardize verbal and non-verbal behavior across interviews. He memorized the three interview scripts, including the IM-tactics to be used. The same standardized interview script was used in every interview condition but the confederate applicant’s IM statements differed per condition so that only entitlements, opinion conformity, or no IM-tactics were used when the confederate applicant (actor) answered the interview questions. The confederate applicant (actor) was dressed in the same, professional way in each of the videotaped interviews, in order not to induce any additional bias (Barrick et al., 2009).

#### Vacancy, Resume, and Interviews

Participants were instructed to read a vacancy for a front-office employee at a fictitious, service-oriented organization (i.e., temporary work agency). The use of IM is considered as natural and even useful for this kind of positions (McFarland et al., 2003; Barrick et al., 2010). Participants were also instructed to carefully read the resume of a 26 years’ old Arab/Moroccan male applicant who was qualified for the job opening (i.e., with relevant educational background and job experiences). The fictitious applicant had done his schooling in Belgium. The same resume was shown in every IM-condition and did not include any IM statements in order not to confound study results (Thoms et al., 1999). Interviews were conducted by the same interviewer who stayed off-screen during the interviews. The interviews were structured: a series of situational and behavioral interview questions (e.g., “Can you give a concrete example of a work situation in which your work planning was turned upside down due to an unexpected event?”) asked about applicants’ job motivation and job relevant competencies. All interviews were recorded in the same professional recording studio under constant lightening conditions and lasted 10 min each.

A small pilot study was conducted prior to the experiment (*n* = 11; 100% ethnic majorities; about 50% of female participants) to evaluate study materials (i.e., applicant; job position; IM-tactics in interviews). First, respondents indicated the ethnic origin of the confederate applicant. Results showed that the applicant was considered from Arab/Moroccan origin, χ^2^(1) = 7.36, *p* < 0.01. Second, participants evaluated the job position on different criteria using a five-point Likert-type scale (1 = *not agree at all* to 5 = *agree very much*). Participants considered the job position as realistic (*M* = 3.91; *SD* = 1.37), clearly formulated (*M* = 4.09; *SD* = 1.37), and high in external client contact (*M* = 4.27; *SD* = 1.27). Third, after having provided definitions on IM-tactics, participants evaluated the IM-tactics used by the confederate applicant in the videotaped interviews using a five-point Likert-type scale. We asked participants whether they perceived any differences across interviews and whether the videos differed from each other in type of IM-tactics used by the confederate applicant (1 = *not agree at all* to 5 = *agree very much*). Results showed that participants perceived clear differences in the way the applicant responded to the interview questions in the three videos (*M* = 4.09; *SD* = 0.94). We further checked whether IM-tactics were perceived as intended. Participants scored the interview in which the confederate applicant used opinion conformity as higher in opinion conformity compared to the two other interviews (*M* = 4.36; *SD* = 1.12). The interview with entitlements was scored higher on entitlements compared to the two other interviews (*M* = 4.27; *SD* = 0.90).

### Measures

After having observed the videotaped interview, participants responded to questions on the following measures, using a five-point Likert-type response scale. *Job suitability* was measured with a four-item measure adapted from Derous et al. (2009). An example item is “How suitable is this candidate for this function based on everything you have seen of him?” (1 = *not suitable at all* to 5 = *very suitable*). Cronbach’s alpha was 0.90. *Ethnic identification* was measured with the Revised Multigroup Ethnic Identity measure (12 items; MEIM-R) of Phinney (1992), Phinney and Ong (2007). An example item is: ‘I have a lot of pride in my ethnic group’ (1 = *strongly disagree* to 5 = s*trongly agree*). Cronbach’s alpha was 0.85. SDO was measured with the Flemish version of the Social Dominance Scale (14 items; Pratto et al., 1994; Van Hiel and Duriez, 2002). An example item is: ‘Some groups of people are simply inferior to other groups’ (1 = *strongly disagree* to 5 = *strongly agree*). Cronbach’s alpha was 0.92. *Interview expertise* was measured in two different ways. We measured participants’ amount of professional experience with interviewing/evaluating candidates as expressed in number of years (1 = 1–12 months, 2 = 1–2 years, 3 = 2–5 years, 4 = 5–10 years, 5 = more than 10 years). We also measured participants’ self-rated interviewing proficiency or perceived expertise with interviewing/evaluating candidates. To this end, we formulated one self-developed item: “How experienced are you in conducting selection interviews, i.e., what is your level of expertise/competence with interviewing?” (1 = *very low*; 5 = *very high*). Finally, participants indicated demographics including their age (1 = younger than 35y; 2 = older than 35y), gender (1 = male, 2 = female), nationality, and ethnic background (“What is your immediate family’s ethnic origin, i.e., yourself, mother and/or father?” followed by different options and an open response field).

## Results

### Preliminary Analyses

Before testing the hypotheses, preliminary analyses were conducted to check manipulations, randomization, correlations, and model assumptions. First, participants read two behavioral examples of IM-tactics (see higher) and indicated whether the applicant used one of these tactics. Manipulation checks showed that minority-preferred IM-tactics, χ^2^(1) = 11.95, *p* < 0.001, and majority-preferred IM-tactics, χ^2^(1) = 18.61, *p* < 0.001, were correctly identified by participants. Also, participants recognized the applicant as being of Arab/Moroccan descent, χ^2^(1) = 86.94, *p* < 0.001. Second, randomization checks showed that experimental conditions did not differ significantly from each other in demographic set-up. Female and male participants were equally distributed across experimental conditions, χ^2^(2) = 0.69, *p* = 0.71, and experimental conditions did not differ from each other in participant age, χ^2^(2) = 0.72, *p* = 0.69. Third, inspection of the correlation table (see **Table 1**) indicated that correlations were not overly strong, except for ‘years’ and ‘self-rated proficiency’ of interview experience (*r* = 0.85) which may pose a threat to collinearity and therefore were not included into the same model. Further, as research has already shown that recruiter and applicant gender might interact with job applicant evaluations due to stereotypes (e.g., Bolino and Turnley, 2003; Tyler and McCullough, 2009; Smith et al., 2013; Waung et al., 2015), and because one’s age might affect how lenient/strict one evaluates others (Wright et al., 2016), we also checked whether raters’ gender and age needed to be controlled for in the main analyses (Berneth and Aguinis, 2016). Participants’ gender and age, however, were not included as they did not appear to be good covariates (i.e., gender and age did not relate to the dependent variable). Other correlations were in line with what could be expected based on previous literature findings. For instance, SDO and ethnic identification correlated significantly positive with each other (*r* = 0.39) but negative with Arab-Moroccan applicants’ job suitability ratings (*r* = -0.20 for ethnic identification and *r* = -0.29 for SDO; e.g., Derous, 2011). Men scored higher on SDO than female respondents (*r* = -0.21; e.g., Pratto et al., 2000). Finally, further exploration of the data indicated that overall multicollinearity was not biasing the model (averaged VIF = 1.01 and tolerance = 0.99; Myers, 1990). Checks on model assumptions showed residuals were independent (Durbin–Watson test = 1.75) and that plots of the standardized residuals against standardized predicted values, histograms and normal PP-plots of the residuals supported the assumptions of homoscedasticity, linearity, and normality.

**Table 1:** Descriptives, correlations, and internal consistencies of study variables.

	*M*	*SD*	*1*	*2*	*3*	*4*	*5*	*6*	*7*
(1) Job suitability	4.00	0.74	*0.90*						
(2) Ethnic identification	2.57	0.43	-0.20^∗∗^	*0.85*					
(3) Social dominance orientation	2.40	0.79	-0.29^∗∗^	0.39^∗∗^	*0.92*				
(4) Interview experience (self-rated interviewing proficiency)	2.40	1.52	-0.25^∗∗^	0.12	0.09	–			
(5) Interview experience (number of years)	2.11	1.39	-0.22^∗^	0.12	-0.02	0.83^∗∗^	–		
(6) Gender^a^	1.72	0.45	0.07	-0.15	-0.21^∗∗^	-0.07	-0.14	–	
(7) Age^a^	1.22	0.42	-0.08	-0.01	-0.10	0.33^∗∗^	0.58^∗∗^	-0.04	–

*Note. Internal consistencies are on the diagonal. ^a^Spearman correlation. Gender: 1 = male; 2 = female; Age: 1 = younger than 35y; 2 = 35y or older*.

^∗^p ≤ 0.05, **p ≤ 0.01.

### Testing of Hypotheses and Research Question

Multiple hierarchical regression analyses with planned orthogonal comparisons were conducted to test the hypotheses and research question. Effects of experimental conditions (i.e., IM-tactics) were entered in a first step, recruiter characteristics were entered as a second step, whereas interaction effects of experimental conditions (i.e., IM-conditions) with recruiter characteristics were entered in a third step (with variables being centered for moderation analyses; Aiken and West, 1991). For the sake of completeness and comprehensiveness, we also considered the predictors’ beta values if these variables were entered in the model at Steps 1 and 2, respectively. These ‘beta in’ values are the standardized coefficients if variables would have been entered into the model in the subsequent stage in a stepwise manner, which gives an idea of the contribution of each of these variables separately into the model, while keeping constant the variables at the first stage (S. Dardha, personal communication, October 18, 2016). **Table 2**, for instance, represents effects of the minority-preferred vs. majority preferred IM-tactics contrast. After Step 1, *R*^2^ was 0.01, *F*(1,155) = 1.50, *p* = 0.22, after Step 2, *R*^2^ was 0.16, *F*(4,152) = 6.95, *p* < 0.001, whereas after Step 3, *R*^2^ was 0.29, *F*(7,149) = 8.63, *p* < 0.001. The adjusted *R*^2^ at Step 3 indicates that more than a quarter of the variability in job suitability ratings is predicted by IM-tactics, recruiter characteristics, and their interactions. **Table 3** further presents a summary of all contrast effects considering the three hypotheses and research question, as further explained below.

**Table 2:** Multiple hierarchical regression analysis predicting job suitability (i.e., hiring outcome) from IM-tactics and recruiter characteristics.

		Job suitability				
		**Step 1**		**Step 2**		**Step 3**
	**Predictors**	**β**	**β In**	**β**	**β In**	**β**

Step 1						
	IM-tactics^a^	0.10		0.14		0.14
Step 2						
	Social dominance orientation		-0.32^∗∗∗^	-0.28^∗∗∗^		-0.20^∗∗^
	Ethnic identification		-0.20^∗∗^	-0.07		-0.04
	Interview experience^b^		-0.33^∗∗∗^	-0.19^∗∗^		-0.21^∗∗^
Step 3						
	IM × Social dominance orientation		0.41^∗∗∗^		0.35^∗∗∗^	0.33^∗∗^
	IM × Ethnic identification		0.18^∗^		0.12	-0.01
	IM × Interview experience		0.20^∗∗^		0.21^∗∗^	0.16^∗^

	Multiple R	0.10		0.40^∗∗∗^		0.54^∗∗∗^
	Total R^2^	0.01		0.16^∗∗∗^		0.29^∗∗∗^
	Adj R^2^	0.00		0.13^∗∗∗^		0.26^∗∗∗^
	ΔR^2^	0.01		0.15^∗∗∗^		0.13^∗∗∗^
	Model *F* (df_1_, df_2_)	1.50 (1,155)		6.95 (4,152)^∗∗∗^		8.63 (7,149)^∗∗∗^

*Note. *N* = 157. ^a^IM (Impression Management): -1 = condition with minority-preferred IM-tactic (entitlements), 0 = control condition (no IM-tactic), 1 = condition with Majority-preferred IM-tactic (opinion conformity). ^b^Interview experience measured as ‘self-rated interviewing proficiency.’ β In = predictor’s beta value if it had been entered into the model in the subsequent stage in a stepwise way. ^∗^*p* ≤ 0.05; ^∗∗^*p* ≤ 0.01; ^∗∗∗^*p* ≤ 0.001*.

**Table 3:** Summary of contrast effects.

	Contrasts	β	*p*-value
Hypothesis 1	Minority-preferred vs. majority preferred IM-tactics	0.10	0.22
	Majority-preferred vs. no IM-tactics	0.02	0.82
	Minority-preferred vs. no IM-tactics	-0.08	0.35
			
Hypothesis 2	Minority-preferred vs. majority preferred IM-tactics	0.33	0.01
	Majority-preferred vs. no IM-tactics	0.18	0.02
	Minority-preferred vs. no IM-tactics	-0.16	0.06
			
Hypothesis 3	Minority-preferred vs. majority preferred IM-tactics	-0.01	0.89
	Majority-preferred vs. no IM-tactics	0.12	0.11
	Minority-preferred vs. no IM-tactics	0.14	0.10
			
Research question	Minority-preferred vs. majority preferred IM-tactics	0.16	0.03
	Majority-preferred vs. no IM-tactics	0.07	0.35
	Minority-preferred vs. no IM-tactics	-0.10	0.18

*Hypothesis 1* expected ethnic majority raters to give lower job suitability ratings to Arab/Moroccan applicants who use minority-preferred IM-tactics or no IM-tactics compared to those who use IM-tactics that are preferred by ethnic majorities. Results, however, showed no significant effect of IM-tactics on job suitability ratings: planned orthogonal contrasts revealed no significant differences between the different IM-tactics’ conditions (minority-preferred vs. majority-preferred IM-tactics, β = 0.10, *p* = 0.22; majority-preferred vs. no IM-tactics, β = 0.02, *p* = 0.82; minority-preferred vs. no IM-tactics, β = -0.08, *p* = 0.35). Participants did not rate the Arab/Moroccan applicant who used entitlements (i.e., minority-preferred IM-tactic) significantly lower (*M* = 3.93; *SD* = 0.90) than when the Arab/Moroccan applicant used opinion conformity (i.e., majority-preferred tactic, *M* = 4.10; *SD* = 0.52), and there were also no differences with the control condition in which no IM-tactics were used (*M* **=** 4.00; *SD* = 0.95). Hypothesis 1, therefore, was not supported.

**FIGURE 1:**
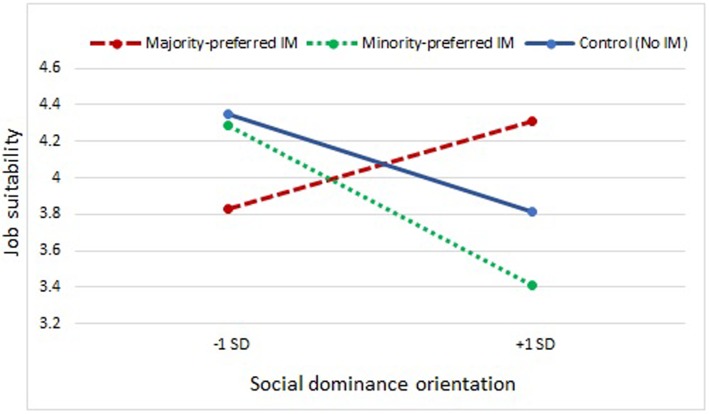
**Moderating effect of social dominance orientation on IM-tactics and interview outcome (job suitability)**.

*Hypotheses 2 and 3* further investigated potential moderating effects of participants’ ingroup/outgroup attitudes (i.e., SDO and ethnic identification) on the relationship between IM-tactics and job suitability ratings. First, hierarchical regressions showed that SDO related negatively to Arab/Moroccan applicants’ job suitability ratings (e.g., Step 3: β = -0.20, *p* = 0.01; see **Table 2**). In support of Hypothesis 2, SDO also moderated the effect of IM-tactics on job suitability ratings. Planned contrasts (see **Table 3**) further showed that SDO moderated the effects of minority-preferred vs. majority-preferred IM-tactics (β = 0.33, *p* = 0.01), and majority-preferred vs. no IM-tactics (β = 0.18, *p* = 0.02), but not the effects of minority-preferred IM-tactics vs. no IM-tactics on job suitability ratings (β = -0.16, *p* = 0.06). When SDO was high, job suitability ratings were significantly lower for the Arab/Moroccan applicant who used minority-preferred IM-tactics (i.e., entitlements) or no IM-tactics as compared to the (same) Arab/Moroccan applicant who used majority-preferred IM-tactics. The opposite pattern was found when recruiters scored low on SDO (see **Figure 1**). Second and contrary to what was expected, raters’ ethnic identification did not relate significantly to job suitability ratings (e.g., Step 3: β = -0.04, *p* = 0.60; see **Table 2**) and did not moderate effects of IM-tactics on job suitability ratings, either (Hypothesis 3; see **Table 3**). Therefore, support was found for Hypothesis 2 on SDO but not for Hypothesis 3 on ethnic identification.

**FIGURE 2:**
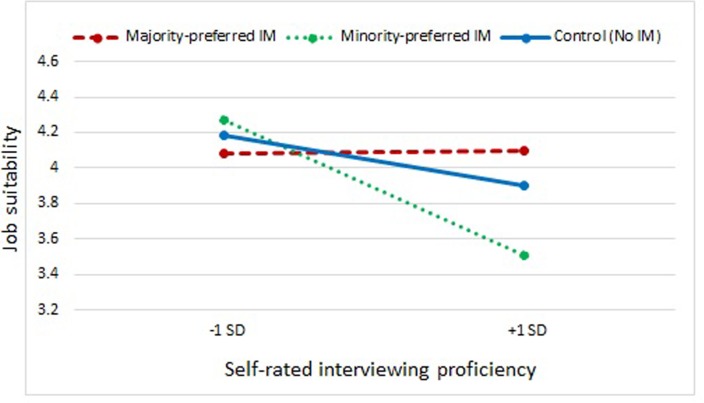
**Moderating effect of interview experience (self-rated interviewing proficiency) on IM-tactics and interview outcome (job suitability)**.

Finally, we formulated a *research question* to explore any potential effects of ethnic majority raters’ professional experience (i.e., self-rated proficiency) in interviewing applicants on Arab/Moroccan minority applicant’s use of IM-tactics and job suitability ratings. For raters’ self-rated proficiency with interviewing^4^, a negative main effect on job suitability ratings (e.g., Step 3: β = -0.21, *p* = 0.01; **Table 2**) was found. Planned contrasts further showed that self-rated interviewing proficiency moderated the effect of minority-preferred vs. majority-preferred IM-tactics (β = 0.16, *p* = 0.03), but not the effects of majority-preferred vs. no IM-tactics (β = 0.07, *p* = 0.35) and also not the effect of minority-preferred IM-tactics vs. no IM-tactics on job suitability ratings (β = -0.10, *p* = 0.18). Raters who scored high on self-rated interviewing proficiency gave significantly lower scores to the Arab/Moroccan applicant who used minority-preferred IM-tactics (entitlements) when compared to the (same) Arab/Moroccan applicant who used majority-preferred IM-tactics (opinion conformity). There were, however, little differences between the IM conditions when self-rated interviewing proficiency was low (see **Figure 2**).

## Discussion

Whereas IM in the employment interview is very well-investigated, the fairness of interview outcomes in relation to how ethnic minorities present themselves remains surprisingly under-researched. That is, studies on IM-tactics rarely took into account applicants’ ethnic origin and sociodemographic background characteristics, which we aimed to address here. Furthermore and contrary to the large amount of studies on decision-making in the social-psychological and organizational literature, research in personnel selection has often failed to consider individual differences in raters’ (i.e., recruiters) tendency to differentiate among candidates. Hence, we also addressed Levashina et al.’s (2014) recommendation to consider more the recruiters’ side when evaluating applicants’ use of IM-tactics in the employment interview.

### Overall Findings

As been postulated by Bilbow (1997), one can expect ethnic minorities to prefer different IM-styles than ethnic majorities. There is indeed evidence for cross-cultural differences in the use of IM-tactics in hiring contexts (Sandal et al., 2014; Fell et al., 2016). These studies, however, showed differential preferences across countries or geographical regions (e.g., Norway vs. Northern-America). Since we do not know previous work that has investigated whether ethnic minority/majority applicants value IM-tactics differently, we first explored whether preferences for the use of IM-tactics depended on applicants’ ethnic-cultural background.

Supporting the central tenet from Bilbow’s model, ethnic minority members of Arab/Moroccan descent had a different preference for ‘assertive’ IM-tactics than ethnic majority members. Both qualitative and quantitative data showed that Arab/Moroccans preferred ‘entitlements’ (i.e., self-focused IM-tactic), whereas Flemish persons favored ‘opinion conformity’ (i.e., other-focused IM-tactic) to a larger extent. At first sight, this may be somewhat surprising: from a theoretical perspective, members from individualistic-oriented societies may be expected to engage more in self-focused behaviors whereas members of collectivistic-oriented societies (like the Arab/Moroccan society), may engage more in other-focused behaviors (Sandal et al., 2014). First, using self-focused IM-tactics (instead of other-focused tactics) might help ethnic minorities to express ‘acculturation’ to Western recruiters, and hence, impress Western recruiters (F. van de Vijver, personal communication, July 3, 2013). Indeed, research has suggested people to try to fit the norms/values of the culture in which they (will) work (Sandal et al., 2014). Second, although Western-European countries are generally higher in individualism than Mediterranean societies, there is also variance among them. For instance, the Netherlands is still higher on individualistic values than its neighboring country Belgium, where modesty is perceived an important value. In Belgium and Flanders in specific, one can voice his/her opinion. Yet, toward power holders a more humble, indirect style is preferred (Hofstede, 2001). Such cultural differences might be very subtle to detect for ethnic minorities when they try to accommodate to the majorities’ cultural norms, specifically in hiring situations. Third, research has also found members of collectivistic cultures not to be consistently low in self-enhancement. Individual differences in modesty as well as socioeconomic situation (König et al., 2012), for instance, have both been suggested to explain differences in self-enhancement and use of IM-tactics. Similarly and in line with findings from König et al. (2012), the overall harsher position of Arab/Moroccan applicants on the Flemish labor market might force applicants to override some cultural norms. Put differently, although Arab/Moroccan minority applicants may generally endorse collectivistic values more than individualistic values, their overall higher unemployment rates might have made them prefer self-focused IM-tactics to other-focused IM-tactics. Taken together, we suggest future research might benefit from measuring ethnic minorities’ level of acculturation and socio-economic situation (Berry, 1997) in addition to applicants’ ethnic background.

Despite the different IM-preferences among both ethnic groups, no differential effects of IM-tactics were found on ethnic minorities’ interview outcomes. If recruiter characteristics were not considered, the use of IM-tactics did not improve the scores of the applicant, also not when compared to the control condition in which no IM-tactics were used. This, however, changed when recruiter characteristics were considered. In particular, recruiters’ SDO and interview experience (i.e., self-rated interviewing proficiency) seemed important boundary conditions.

First, the higher raters scored on SDO, the lower they evaluated the Arab/Moroccan applicant, specifically when the applicant used entitlements (i.e., Arab/Moroccan-preferred IM-tactic) as compared to opinion conformity (i.e., Flemish/Belgian-preferred IM-tactic). These findings corroborate with predictions from social identity (Tajfel and Turner, 1986) and social dominance theories (Sidanius and Pratto, 1999): minorities who strongly identify with their ethnic ingroup may be perceived as more threatening and challenging by majority raters that are high in SDO. Findings mirror earlier results from Umphress et al. (2007) that showed that prospective employees from high-status groups (ethnic majorities) showed ingroup favoritism if they supported group-based social hierarchies. In a hiring scenario, Derous (2011) further showed ethnic majority raters’ SDO to negatively affect resume scores of highly ethnically identified Arab applicants but not those of equally qualified native Dutch applicants. Interestingly, our study findings do not support the ‘status boundary enforcement hypothesis’ (Thomson et al., 2008), which would predict more prejudice from high social dominators against ethnic minorities who assimilate into the dominant culture (e.g., by using opinion conformity). Such applicants might be perceived as ‘blurring the status boundaries’ and challenging the dominant hierarchy. According to the status boundary enforcement hypothesis this would be particularly the case for ethnic minorities that are perceived as members of a very distinct group that tries to infiltrate into the host culture. First, as Arab/Moroccan immigration started about 50 years ago (Schoonvaere, 2014) and because our job applicant spoke Flemish (fluently) and had studied in Belgium, it is rather unlikely one would have considered the applicant as a member of a ‘very distinct group that is trying to infiltrate in Flemish/Belgian society.’ Further research, however, could test whether a different pattern emerges for relatively new incoming and more distinct groups of ethnic minorities (such as Middle Eastern refugees) due to recent political and humanitarian crises (like the Syria crisis). That is, high dominators could perceive relatively new groups of migrants who assimilate in a very explicit way as challenging the status boundaries of ethnic majority groups to a larger extent than migrant groups that already reside in a host country for some time and have shown some patterns of assimilation. Second, there is considerable empirical evidence showing that members of dominant, ethnic majority groups in Western-Europe have adopted assimilation ideologies (e.g., ‘melting pot’), meaning that ethnic majorities reveal more positive attitudes toward assimilation than toward multiculturalism (e.g., Verkuyten, 2005, 2007; Van Oudenhoven et al., 2006). Indeed and in line with findings from acculturation studies (e.g., Verkuyten, 2007), stressing a common ethnic background by using majority-preferred IM-tactics led to *higher* job suitability ratings by those who scored rather high on SDO, whereas stressing a different ethnic background by using minority-preferred IM-tactics led to *lower* job suitability ratings of the same ethnic minority candidate. Hence, study findings also support previous evidence that multiculturalism is perceived as more threatening to ethnic majorities than assimilation ideologies, which is consistent with both social identity and social dominance theories (Guimond et al., 2010).

Second, we expected ethnic majority raters high in ethnic identification to give lower job suitability ratings to ethnic minority members who used minority-preferred IM-tactics than to those who used majority-preferred IM-tactics. The ‘beta included’ variables column (see **Table 2**) as well as additional hierarchical regression analyses^5^ showed a significant negative effect for ethnic identification, but *only so* when recruiters’ SDO was *not* (yet) included in the model. That is, the more recruiters identified with their ethnic ingroup (without consideration of their actual level of SDO), the lower they scored Arab/Moroccan applicants’ job suitability ratings, which is in line with predictions from the social identity theory. However, this effect became non-significant when raters’ level of SDO were taken into account: social dominators who identify with their ethnic group and dominant social position in society, will protect the status of their ingroup and engage in outgroup derogation, which may explain the diminished value of raters’ ethnic identification on ethnic minority applicants’ evaluations. Although this was not the goal of the present study, further research could delve more into, e.g., the potential mediating role of SDO regarding ethnic majority recruiters’ identification with their dominant ethnic ingroup and their evaluation of ethnic minority applicants (Guimond et al., 2003; Shook et al., 2016).

Third, we also explored any potential relationship between ethnic majority recruiters’ interview experience and their evaluation of Arab/Moroccan applicants’ use of IM-tactics. Overall, more experienced raters (as operationalized by self-rated interviewing proficiency) gave lower ratings than their less experienced counterparts. However, experienced raters were also more prone to biased decision-making against an ethnic minority applicant who used entitlements, when compared to an equally qualified ethnic minority applicant who used opinion conformity. Once again, recruiters could have perceived applicants who used entitlements as less well-assimilated than those who used opinion conformity. Alternatively, less experienced recruiters (as operationalized by self-rated interviewing proficiency) might have been less prejudiced compared to more experienced raters. *Post hoc* tests, however, did not show significant interactions of self-rated interviewing experience with raters’ ingroup/outgroup attitudes on interview ratings. As yet another alternative explanation, more experienced raters (i.e., self-rated proficiency) might have been more able to detect differences between IM-tactics (or not using any IM-tactics) than their less experienced counterparts (Delery and Kacmar, 1998). Indeed, less experienced raters gave overall higher scores and scores did not differ between the three conditions of IM-tactics. Perhaps less experienced majority raters (as operationalized by self-rated interviewing proficiency) are less sensitive to different IM-tactics employed by the ethnic minority applicant. Or contrarily, perhaps they may have used *more* elaborated processing of stigmatizing applicant information compared to their more experienced counterparts, hence explaining the absence of bias (Derous et al., 2016). More research is needed to investigate mechanisms of (self-rated) interview experience to a further extent, as this remains relatively underinvestigated in the interview literature.

Note that we investigated ‘self-rated interviewing proficiency’ instead of ‘quantity’ of interview experience (i.e., years of interview experience) as is typically done (e.g., Dipboye and Jackson, 1999) but which may not reflect the perceived quality or specificity of interview experience. However, as with any type of work experience (Tesluk and Jacobs, 1998), recruiters’ self-rated interviewing proficiency can also be considered to be more complex and multidimensional as measured here. For instance, having more specific experiences with interviewing ethnic minority applicants may make recruiters more familiar with minority-preferred IM-tactics and behavior, which could counter prejudiced decision-making. Furthermore, as both self-rated interviewing proficiency and years of interview experience were highly intercorrelated (*r* = 0.83), a more objective measure of interviewing proficiency (e.g., by using other ratings) could also be considered. Hence, we suggest future research might take an even more fine-grained perspective on effects of both quantitative and qualitative interview experiences (Dipboye and Jackson, 1999).

Finally, interview evaluations of minority applicants who used no IM-tactics did not differ much from minority-preferred IM-tactics, also not when recruiters’ characteristics were taken into account (**Table 3**). Maybe not using any IM-tactic might also signal lower levels of assimilation, thereby stressing minority applicants’ outgroup status in a similar way as when minority-preferred IM-tactics are used. Interestingly, whereas self-focused IM-tactics (like entitlements) have generally been considered as effective strategies, our data showed effectiveness to depend on both ethnic minority background of the applicant and recruiter characteristics. Hence, the effectiveness of IM-tactics might be contingent upon the particular context in which tactics are used. These findings underscore earlier findings stressing the importance of recruiters’ perceptions of applicant IM-tactics (Roulin et al., 2014).

### Strengths, Limitations, and Research Implications

We agree with Levashina et al. (2014) that research on IM in structured interviews is still a relatively underdeveloped area. This especially counts for cultural effects of IM-tactics on ethnic majority recruiters’ perceptions and interview evaluations (Dipboye et al., 2013; Tsai and Huang, 2013), which we started exploring here. Our study also adds to the existing literature in several other ways. First, we considered one particular selection tool (i.e., the employment interview) instead of investigating IM during the selection procedure (in general; e.g., König et al., 2011). Second, most studies on cross-cultural IM-tactics have used student samples. Instead of focusing on differences in students’ self-reported IM-tactics using cross-sectional survey methods (e.g., Bye et al., 2011; Sandal et al., 2014), we used an experiment to evaluate how recruiters assessed different IM-tactics. Third, by also investigating recruiter characteristics, we started studying boundary conditions that might explain why/when IM-tactics might affect interview outcomes (Levashina et al., 2014; Roulin et al., 2014; Bolino et al., 2016). Finally, whereas previous studies compared self-reported IM-tactics across countries (e.g., North-American vs. China; Sandal and Endresen, 2002; König et al., 2012; Fell et al., 2016), we considered actual IM-tactics as employed by ethnic minorities on ethnic majorities’ evaluation of the minority applicant within the same ‘host’ country.

As with any study, however, limitations need to be mentioned. First, we carefully investigated IM-tactics in a preliminary study and subsequently developed study materials for our experimental study, based on these findings. We proceeded in both an inductive and deductive way by using existing IM-taxonomies to evaluate observed IM-tactics. Such taxonomies have been applied successfully in other cultures too (e.g., König et al., 2011; Fell et al., 2016), despite the fact that most IM-taxonomies reflect Western values and norms (where they were developed) and do not necessarily apply abroad (i.e., ‘cultural relativism’). Preferences for IM-tactics emerged from our preliminary study (i.e., focus group and survey): both focus group and survey data showed that Arab/Moroccan persons preferred entitlements whereas Flemish/Belgian persons preferred opinion conformity (effect sizes were moderate to large). Therefore, in the experimental study we did not cross-check Flemish recruiters’ *preferences for IM-tactics* anymore. However, it could have been possible that recruiters had other preferences than our preliminary study findings indicated. Such an alternative explanation cannot be excluded for 100% but seems rather unlikely given the preliminary findings and given that we randomized recruiters across conditions. Additional randomization checks, for instance, showed that our three conditions did not differ significantly regarding level of recruiters’ SDO, ethnic identification, and professional experience (both in terms of self-rated proficiency and number of years of professional experience). Nevertheless, future research might attempt to measure recruiters’ preferences for IM-tactics in a more direct way to cross-check findings from pilot tests and/or preliminary studies. In addition, one might also consider other individual differences (like ethnic minorities’ level of acculturation) that might moderate such effects.

Second, only two *types of verbal IM-behav*ior (i.e., one type per condition) were manipulated in the experimental study. In actual employment interviews, however, applicants may engage in more than one verbal IM-tactic at a time (Proost et al., 2010), which could counter recruiters’ ethnic biases, e.g., when ethnic minority applicants use both minority and majority-preferred IM-tactics. Research could test this. Furthermore, applicants may also use non-verbal IM-tactics (like eye contact) that may signal their ethnic outgroup status to a larger (or lesser) extent than when only verbal IM-tactics are considered. Whether non-verbal IM-tactics are considered appropriate may also depend on sociocultural norms of the majority culture (Bilbow, 1997) and is subject to investigation. Hence, the potential differential influence of ‘culture-specific’ verbal vs. non-verbal IM-tactics on interview ratings, may be an interesting avenue for further research.

Third, for practical reasons we measured *recruiter characteristics* (like SDO) immediately after recruiters saw the applicant video, which might have affected how participants responded to these measures (Guimond et al., 2003). Additional analyses, however, showed no differential effect of IM-tactics on participants’ reported SDO, *F*(1,161) = 1.59, *p* = 0.21, and also not on their level of ethnic identification, *F*(1,155) = 0.10, *p* = 0.75. Hence, SDO and ethnic identification were not affected by the experimental manipulation. Although challenging, further research could consider a two-phased experimental study in which recruiter characteristics are measured sometime before the interview and/or one could consider the use of distractor tasks in between both measurements (e.g., Podsakoff et al., 2003).

Fourth, Bolino et al. (2016) plead for more research on IM in *specific cultures*. Although Arab/Moroccan job seekers are a very specific and relevant population to study (i.e., given their overall more precarious labor market position in many Western-European countries), our findings are restricted to only one ethnic minority group in one particular country. Follow-up studies might benefit from investigating other ethnic minority groups and labor markets to see whether overall findings hold and can be generalized. Furthermore, whether ethnic majority applicants would be evaluated differently depending on type of IM-tactic was not the goal of the present study. Although challenging, testing a model in which ethnic minority vs. majority-preferred IM-tactics are crossed with ethnic minority/majority status of both applicants and recruiters would allow to draw more firm conclusions about the overall efficiency of IM-tactics as a function of applicants and recruiters’ ethnic background.

Finally, the *scenario-based* nature of our study has its strengths but limitations too. Given that employment interviews are two-way processes in which both the recruiter and applicant influence each other, one could assume applicants’ use of IM-tactics to evolve during the interview. Indeed, also IM is considered as a two-way process (Bilbow, 1997). That is, recruiters also use IM-tactics and/or may signal (explicit or subtle) IM preferences (Stevens et al., 1990; Wilhelmy et al., 2016). Applicants can pick-up such signals and change tactics accordingly to increase recruiters’ fit perceptions during the course of the interview (Roulin et al., 2016). Whether ethnic minority applicants, for instance, may reciprocate ethnic majority recruiters by mimicking their IM-tactics to induce ‘similar-to-me effects’ (Liden et al., 1993) and increase their job chances, is another interesting avenue for further research. We feel these more dynamic aspects of the use of IM-tactics need more research attention in general and in the context of cross-cultural employment interviews, in particular. In line with our focus group approach, real interviews could be investigated using observational designs or videotaped interviews, that also consider dynamic patterns and mutual effects of ethnic majority recruiters and ethnic minority applicants’ use of IM-tactics.

### Conclusion

This study investigated whether ethnic minorities’ use of IM-tactics, aimed to boost interview evaluations, could also negatively affect their interview ratings as made by ethnic majority members. Overall, majority-preferred IM-tactics seemed more helpful to ethnic minorities than minority-preferred IM-tactics but this effect depended entirely on majority recruiters’ characteristics. Study findings, therefore, might provide some practical guidance on the training of recruiters in order to help them overcome biased decision-making. One puzzling outcome, for instance, pertains to the negative effect of recruiters’ (self-rated) professional experience. As higher quality judgments might depend on the knowledge structures that facilitate processing of applicant information (Dipboye and Jackson, 1999), one may educate interviewers about cultural differences in IM-tactics and spontaneous Type 1 processing mechanisms to avoid biased decision-making (Derous et al., 2016).

Somewhat more difficult to train, are recruiters prejudiced ingroup/outgroup attitudes, like SDO. Perhaps the screening of recruiters on prejudiced attitudes could be helpful in this regard. Recently, however, Shook et al. (2016) showed that significant intergroup contact resulted into lower levels of SDO and more positive attitudes toward racial/ethnic groups. Yet, whether such training effects occur in other settings (like recruitment and selection) has yet to be proven. Note that the biasing effects in our study also occurred when a structured interview format was used. One alternative, therefore, could be the use of demographically diverse panels of interviewers instead of one-to-one interviews (which may reduce bias but may be less practical to organize). Holding interviewers accountable for their selection decisions may be another interesting strategy to explore (e.g., Self et al., 2015). Finally, study findings may also be valuable to coach and educate ethnic minority job seekers about potential misinterpretations when (not) using certain IM-tactics as some tactics might be considered more (or less) appropriate than others (Spong and Kamau, 2012). Such insights may also be helpful for international selection (e.g., expatriates; Mendenhall and Wiley, 1994). It further helps maximizing qualified ethnic minorities’ job chances and combat hiring discrimination, which ultimately may result in a competitive advantage for organizations, given the war for talent that many organizations experience these days.

## Author Contributions

The author formulated the research project, supervised/guided the data collection of the project, and wrote the manuscript.

## Conflict of Interest Statement

The author declares that the research was conducted in the absence of any commercial or financial relationships that could be construed as a potential conflict of interest. The handling Editor declared a past collaboration with the author and states that the process nevertheless met the standards of a fair and objective review.

## Footnotes

1 Nomenclature: IM is the abbreviation of impression management. CCIM, Cross Cultural Impression Management Discourse model; SDO, social dominance orientation.

2 Although that applicants’ sociocultural background and ethnic minority status are to a certain extent interrelated, the focus in this study is on cultural differences in IM-tactics rather than differences due to the minority status of the applicant.

3 Because no previous studies have tested these assumptions in these particular groups, no specific hypotheses were formulated.

4 As interview experience has also been expressed in number of years in the literature (Dipboye and Jackson, 1999), we tested an additional model that included raters’ years of experience instead of self-rated interviewing proficiency (Note that due to issues with multicollinearity, self-rated proficiency and amount of years of interview experience were tested in different models instead of one model). Results showed a significant negative effect of years of experience on job suitability ratings (β = -0.24, *p* = 0.00) and a marginally significant interaction effect with IM-tactics (β = 0.14, *p* = 0.05).

5 Additional hierarchical regressions in which job suitability was regressed on IM-tactics (entered in a first step), ethnic identification (entered in the second step), and social dominance orientation (entered in the third step) showed also that ethnic identification became non-significant when social dominance orientation was considered.
